# Hollow Abutment Screw Design for Easy Retrieval in Case of Screw Fracture in Dental Implant System

**DOI:** 10.1155/2017/4842072

**Published:** 2017-08-06

**Authors:** Bo Kyun Sim, Bongju Kim, Min Jeong Kim, Guk Hyun Jeong, Kyung Won Ju, Yoo Jin Shin, Man Yong Kim, Jong-Ho Lee

**Affiliations:** ^1^Clinical Translational Research Center for Dental Science, Seoul National University Dental Hospital, Seoul, Republic of Korea; ^2^Department of Oral and Maxillofacial Surgery, School of Dentistry, Seoul National University, Seoul, Republic of Korea

## Abstract

The prosthetic component of dental implant is attached on the abutment which is connected to the fixture with an abutment screw. The abutment screw fracture is not frequent; however, the retrieval of the fractured screw is not easy, and it poses complications. A retrieval kit was developed which utilizes screw removal drills to make a hole on the fractured screw that provides an engaging drill to unscrew it. To minimize this process, the abutment screw is modified with a prefabricated access hole for easy retrieval. This study aimed to introduce this modified design of the abutment screw, the concept of easy retrieval, and to compare the mechanical strengths of the conventional and hollow abutment screws by finite element analysis (FEA) and mechanical test. In the FEA results, both types of abutment screws showed similar stress distribution in the single artificial tooth system. A maximum load difference of about 2% occurred in the vertical load by a mechanical test. This study showed that the hollow abutment screw may be an alternative to the conventional abutment screws because this is designed for easy retrieval and that both abutment screws showed no significant difference in the mechanical tests and in the FEA.

## 1. Introduction

Placement of dental implants has become the main treatment option for oral function recovery in partially or completely edentulous patients. The components of a dental implant consist of fixture, abutment, and abutment screws.

Despite the high success rate of implants, it is not free of complications and dental implants occasionally fail due to biological factors or technical complications [1, 2]. The technical problems of implant-based restoration components including abutment screw fracture and peri-implantitis are deeply related to dental implant system failure, and an increase in related complications are also being reported [3–5]. Many studies have reported that after osseointegration of the implant, abutment screw loosening and fracture are the most common problems, and other mechanical problems involve prosthesis fracture and overdenture attachments [6, 7]. One study reported that the incidence rate of screw fracture is 3.9% which is normally due to overload or elevated torque [6, 8]. If the abutment screw fractures, the screw must be removed and replaced with a new one so that implant prosthesis may be fabricated again. Otherwise, it will compromise the long-term success of the implant [9].

Majority of implant failures nowadays are caused by mechanical factors rather than the implant itself, and so, there are alternative abutment systems that were developed. Only a few studies on the removal of fractured screw in the implant [10, 11] were reported. Many techniques and methods were shown through case reports which all concluded that removing the fractured screw from an implant can be difficult and that there is no universal method that can be applied. Therefore, the abutment screw was modified to have a prefabricated access hole for easy retrieval when the abutment screw fractures. The purpose of this study was to introduce this modified design of the abutment screw, the concept for easy retrieval with this new innovative design, and to compare the mechanical strengths of the conventional abutment screws and the modified version with prefabricated access holes.

## 2. Materials and Method

### 2.1. Hollow Abutment Screw Design for Easy Retrieval

**Figure 1:**
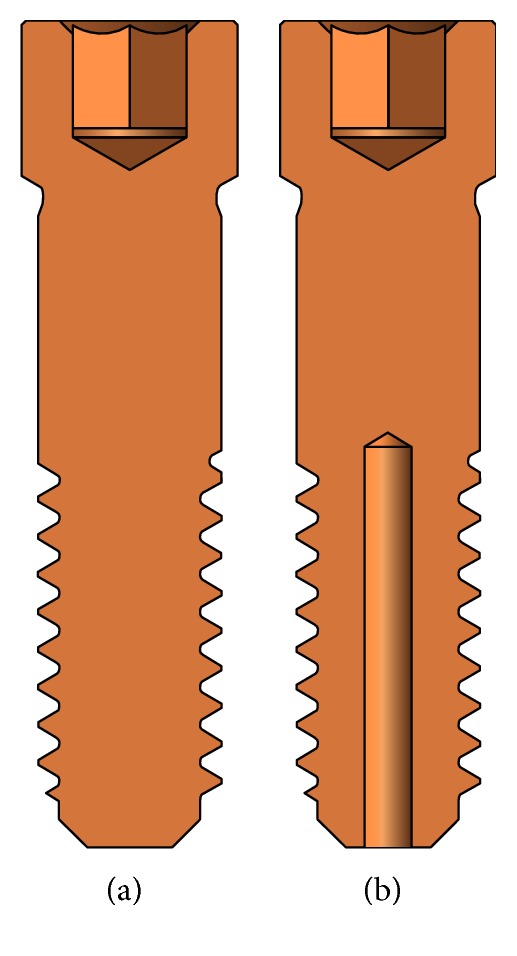
Abutment screw design. (a) Conventional type. (b) Hollow type.

Conventional abutment screw measures 2 mm in diameter, 7.8 mm in length, and 0.4 mm in screw thread pitch (Figure 1()), and the hollow abutment screw was made with the same system as the conventional abutment screw but modified by creating a hole of 0.5 mm in diameter from the lower end of the abutment screw up to the 1st thread (Figure 1(b)).

For easy retrieval of the fractured screw of the hollow type screw (modified with prefabricated access hole), the hole was fabricated with a reverse screw drill. The reversed screw was tightened with the screw driver allowing stabilization of the fractured portion, and the external thread would be unscrewed from the fixture.

### 2.2. Mechanical Test and Finite Element Analysis

3D implant system models were constructed using Solidworks 2016 (Dassault Systèmes) for this study. Mechanical test was performed on the conventional abutment screw and the hollow abutment screw by fabricating 3D models, and finite element analysis (FEA) was implemented to the single artificial tooth system model.

**Figure 2:**
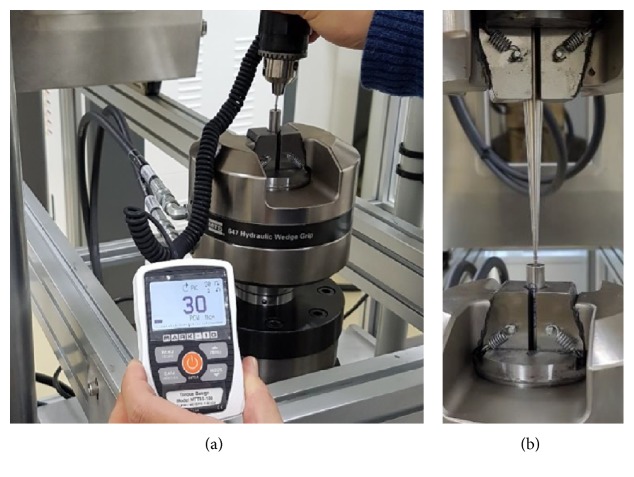
Example of mechanical test. (a) Fixed abutment screw on the jig by applying insertion torque. (b) Vertical loading.

MTS Bionix 370.02 (MTS Systems Co., USA) was used for the vertical load test to compare the mechanical strength of the conventional abutment screw and the hollow abutment screw. Before the vertical load test, the abutment screw was fixed on the jig vertically by applying 30 N·cm of insertion torque (Figure 2(a)) [12]. The experiment was performed with a load speed of 5 mm per minute where it was fixed on the equipment (Figure 2(b), *N* = 3, independent experiments).

**Figure 3:**
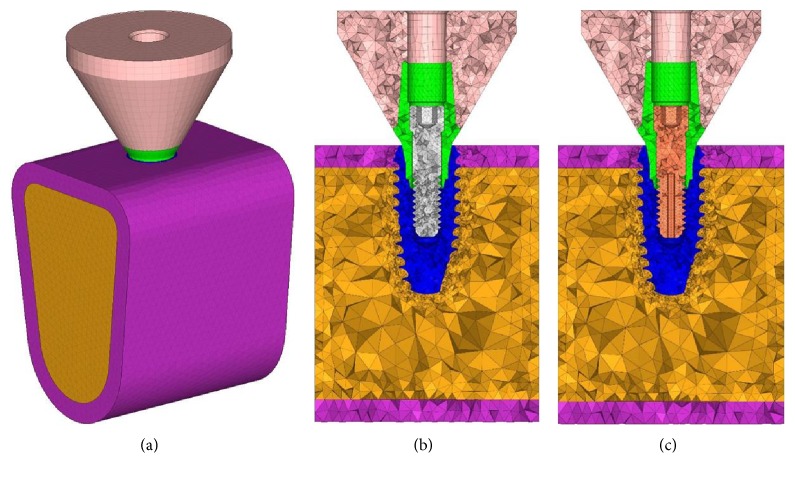
3D finite element model. (a) Constructed 3D model, (b) conventional type, and (c) hollow type.

IS II Active Implant System (NeoBiotech Co., Korea) was used in fabricating the 3D model of the single implant system design (Figure 1). The bone model height is 17 mm, the width is 30 mm, and bucco-lingual thickness is 13 mm and consisted of a 1.5 mm layer of the cortical region. The height of the crown is 9 mm with the diameter of 12 mm, and it was modeled in a flat form (Figure 3).

Hypermesh version 14 (Altair Engineering Inc., USA) was used to construct the model for FEA (Figure 3), and FEA was performed using Abaqus 6.16 (Dassault Systèmes, France).

The values assumed for the modulus of elasticity and Poisson's ratio are given in Table 1, and the nodes and elements are shown in Table 2. All materials used in the models were considered to be isotropic, homogenous, and linear elastic. Boundary fixation included restraints for all six degrees of freedom including rotation and translation in three coordinate axes for the correspondent nodes located at the bottom and both sides of the bone model including the cancellous bone. Various sizes of masticatory force are being reported; however, this study conducted FEA on two types of load (500 N vertical load and 142 N horizontal load) with the concentrated load on the center of the upper artificial prosthesis [13].

### 2.3. Statistical Analysis

Sample sizes were estimated for comparison of two groups based on previous study (total sample sizes = 7, [14]). The experimental result was based on three repeated measurements under the same loading condition independently (1 set, conventional, and hollow type, *n* = 3, resp.).

All data are presented as means ± SD of independent recordings. Statistical analyses were performed by unpaired two-tailed *t*-test (SigmaPlot, Systat Software Inc., San Jose, CA, USA). *P* < 0.05 was considered significant.

## 3. Results and Discussion

**Figure 4:**
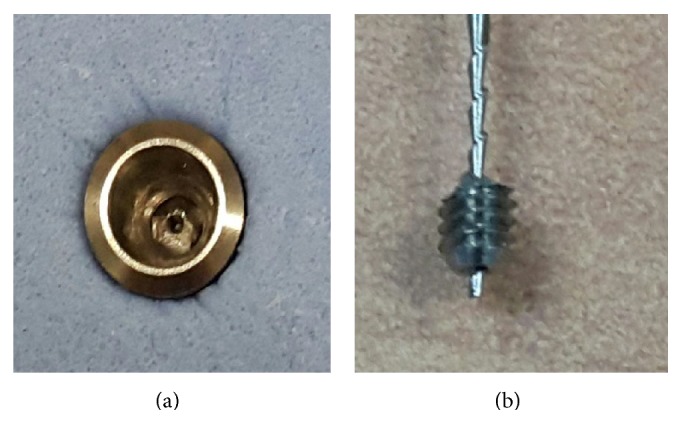
(a) Abutment screw fracture on the hollow abutment screw. (b) Hollow abutment screw is easily retrieved with the H-file.

Hollow abutment screw eliminates the usage of a commercially available screw retrieval kit which was developed to utilize screw removal drills to make a hole on the fractured screw which provides an engaging drill to unscrew it. Hence, the fractured screw could be easily removed using the H-file (Figure 4).

**Figure 5:**
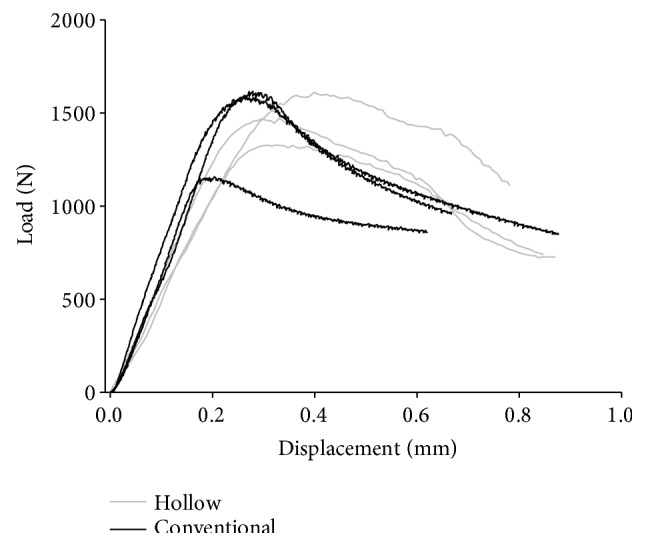
Load-displacement curve of abutment screws.

**Figure 6:**
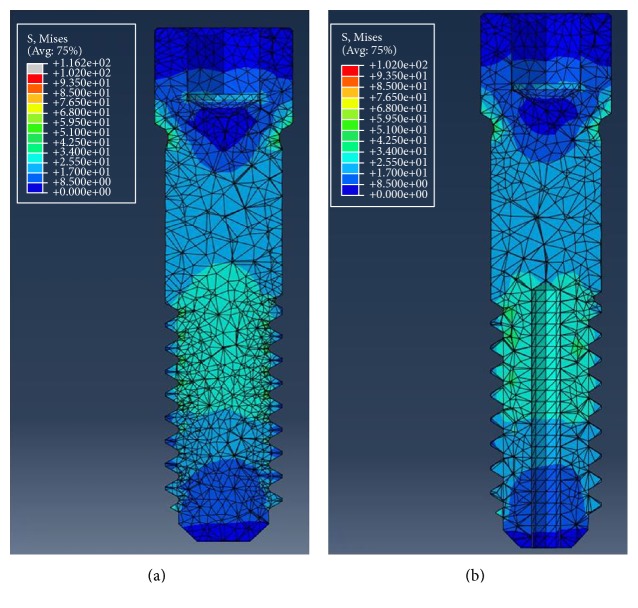
von Mises stress distribution under vertical loading condition. (a) Conventional abutment screw. (b) Hollow abutment screw.

**Figure 7:**
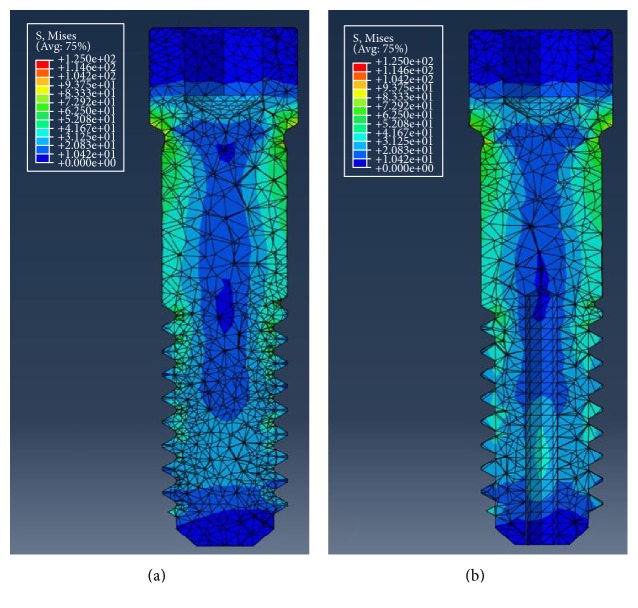
von Mises stress distribution under horizontal loading condition. (a) Conventional abutment screw. (b) Hollow abutment screw.

In the vertical load mechanical test, maximum compressive load did not show significant difference between the conventional and the hollow abutment screws (Table 3, Figure 5).  Table 4 shows the maximum von Mises stress occurred in the abutment screws of each type by vertical and horizontal load in a single artificial tooth system. For the vertical load, approximately 19.68 MPa higher von Mises stress value was observed in the conventional abutment screw, where the value was 116.16 MPa for the conventional type and 96.48 MPa for the hollow type. Both the conventional and hollow abutment screws showed similar stress distribution in the single artificial teeth system (Figure 6). For the horizontal load, von Mises stress value was higher in the hollow type screw by approximately 4.66 MPa, where the value was 110.99 MPa for the conventional type screw and 106.33 MPa for the hollow type screw. Similar stress distribution was observed as well as for the horizontal load (Figure 7).

As this experiment does not provide a standard for strength evaluation of the abutment screw, it was performed according to the ASTM standard (ASTM F543—standard specification and test methods for metallic medical bone screws) on metallic bone screw, and the experiment was performed with only the abutment screw. Thereafter, strength evaluation of the conventional and the hollow abutment screws was performed with a mechanical test, and the maximum load difference of about 2% occurred in the vertical load.

In the mechanical test of the three specimens, there were differences depending on the specimens' processing conditions. However, all three specimens showed similar load form, and it was considered that not much difference was observed in the conventional and hollow abutment screws.

In this study, the compressive strength test and FEA were performed to compare the physical performance of the conventional type and the hollow type abutment screws. However, since the physical performance on fatigue loading is important, it is necessary to test and verify the fatigue of the screw in future studies in the actual implant system.

## 4. Conclusion

Management of fractured abutment screw is clinically challenging and timely but it is necessary to provide an adequate rehabilitation plan. This study showed that the hollow abutment screw may be an alternative to the conventional abutment screws because this is designed for easy retrieval and that both abutment screws showed no significant difference in the mechanical tests and in FEA.

## Figures and Tables

**Table 1:** Material properties.

Components	Material	Elasticity (Gpa)	Poisson's ratio
Crown	Zirconia	260	0.28
Abutment	Ti alloy	113.8	0.342
Fixture			
Abutment screw			
Cortical bone	Cortical bone	14.0	0.3
Cancellous bone	Cancellous bone	1.5	0.45

**Table 2:** Number of elements and nodes.

Components	Elements		Nodes	
	Conventional	Hollow	Conventional	Hollow
Crown	29,614		6441	
Abutment	6242		1868	
Fixture	5441		13,307	
Cortical bone	32,916		8633	
Cancellous bone	149,721		31,303	
Abutment screw	26,792	14,513	5963	3667

**Table 3:** Mechanical test parameters of two different types of abutment screws, *n* = 9, respectively.

Direction	Type	Value	Max load (N)	Displacement at max load (mm)
Vertical	Conventional	Ave.	1452.27	0.25
		SD	256.10	0.04
	Hollow	Ave.	1480.37	0.35
		SD	137.90	0.05

No significant difference between the two groups (*P* > 0.05).

**Table 4:** Finite element analysis results of two different types of abutment screw forms: vertical load and horizontal load.

Load	Type	von Mises stress (MPa)
Vertical	Conventional	116.16
	Hollow	96.48
Horizontal	Conventional	110.99
	Hollow	106.33
